# Investigating the effect of an online enhanced care program on the emotional and physical wellbeing of patients discharged from hospital with acute decompensated heart failure: Study protocol for a randomised controlled trial: Enhanced care program for heart failure

**DOI:** 10.1177/20552076241256503

**Published:** 2024-05-28

**Authors:** Kristy Fakes, Breanne Hobden, Nick Zwar, Nick Collins, Christopher Oldmeadow, Francesco Paolucci, Allan Davies, Irosh Fernando, Michael McGee, Trent Williams, Cameron Robson, Robert Hungerford, Jia Ying Ooi, Aaron L Sverdlov, Rob Sanson-Fisher, Andrew J Boyle

**Affiliations:** 1 Health Behaviour Research Collaborative, School of Medicine and Public Health, College of Health, Medicine and Wellbeing, 5982 University of Newcastle, Callaghan, New South Wales, Australia; 2 454568 Hunter Medical Research Institute, New Lambton Heights, New South Wales, Australia; 3 3555 Bond University, Faculty of Health Sciences and Medicine, Robina, Queensland, Australia; 4 37024 Cardiovascular Department, John Hunter Hospital, New Lambton Heights, New South Wales, Australia; 5 Newcastle Business School, University of Newcastle, Callaghan, New South Wales, Australia; 6 School of Medicine and Public Health, College of Health, Medicine and Wellbeing, University of Newcastle, Callaghan, New South Wales, Australia; 7 Department of Sociology, Law and Economics, 9296 University of Bologna Bologna BO, Italy; 8 Newcastle Mental Health Service, Hunter New England Local Area Health District, New South Wales, Newcastle, Australia; 9 72540 Cardiac Department, Tamworth Rural Referral Hospital, North Tamworth New South Wales, Australia; 10 School of Nursing and Midwifery, College of Health, Medicine and Wellbeing, University of Newcastle, Callaghan, New South Wales, Australia

**Keywords:** (MeSH terms): heart failure, depression, quality of life, internet-based intervention, randomised controlled trial

## Abstract

**Objective:**

Depression is highly prevalent and associated with increased hospitalisations and mortality among patients with heart failure (HF). This study will evaluate the effectiveness and cost-effectiveness of an online wellbeing program for patients discharged from hospital with acute decompensated heart failure (ADHF) in (i) improving emotional and physical wellbeing, and (ii) decreasing healthcare utilisation.

**Methods:**

Two-arm randomised controlled trial. Eligible patients with ADHF will be recruited pre-discharge from two hospitals. Five hundred and seventy participants will be randomised to receive the intervention (online enhanced care program for HF: ‘Enhanced HF Care’) or usual care. Enhanced HF Care includes health education (11 micro-learning modules) and monitoring of depression and clinical outcomes via fortnightly/monthly surveys for 6 months, with participants offered tailored advice via video email and SMS. Cardiac nurses track real-time patient data from a dashboard and receive automated email alerts when patients report medium- or high-risk levels of depression or clinical symptoms, to action where needed. General practitioners also receive automated alerts if patients report medium- or high-risk survey responses and are encouraged to schedule a patient consultation.

**Results:**

Sixty-five participants enrolled to-date. Co-primary outcomes (‘Minnesota Living with Heart Failure Questionnaire’ Emotional and Physical subscales) and healthcare utilisation (secondary outcome) at 1- and 6-month post-recruitment will be compared between treatment arms using linear mixed effects regression models.

**Conclusions:**

This study has the potential to reduce the burden of depression for patients with HF by prioritising urgent mental health needs and clinical symptoms while simultaneously empowering patients with self-care knowledge.

**Trial registration:**

The trial was prospectively registered via the Australian New Zealand Clinical Trials Registry: ACTRN12622001289707. Issue date: 4 October 2022.

## Introduction

Heart failure (HF) is a debilitating and economically burdensome condition,^1^ estimated to impact over 64 million people globally,^2^ with costly hospitalisations and high comorbidity and mortality.^3^ Acute decompensated heart failure (ADHF), the worsening of symptoms of HF, is a frequent cause of hospitalization^4^ and patients experience poor outcomes. In addition to significant mortality rates, there have also been several gaps demonstrated in patient care for emotional wellbeing.^5,6^ Almost 50% of patients with HF have depressive symptoms and approximately one-third have symptoms of anxiety.^7^ Depression has been associated with increased hospital utilisation, reduced health functioning and a higher risk of all-cause mortality.^8^ Given the interaction between emotional wellbeing and physical outcomes including disease progression,^9^ as well as the importance of patients’ mental health, there is a need for increased emotional support and information for patients with HF.

The post-diagnosis and post-discharge periods can be an overwhelming and anxious time for patients, and this anxiety is associated with reduced retention of information.^10^ Previous data also indicates that when patients with HF are experiencing depressive symptoms, these symptoms are unrecognised by providers in 50% of cases.^11^ Follow-up community care is typically nurse-led but varies in length and intensity. Innovative models are required for improving discharge support and community care, which harness existing community resources, such as general practitioners (GPs), and increases the probability of a care partnership between healthcare providers, patients, and their support persons.

Consistent focused post-discharge community support may offer significant opportunity to improve emotional and physical wellbeing of patients with HF. Secondary prevention including the identification and management of modifiable health risk factors, psychosocial care, education, and support for self-management is also important and guideline recommended.^12^ For example, preventive strategies such as exercise have been shown to have positive effects on cardiovascular disease,^13^ including patients with HF,^14^ and individuals with risk factors for cardiovascular disease, such as type 2 diabetes and obesity.^15,16^ Digital health interventions represent a potentially effective mechanism for helping patients with HF manage their emotional and physical wellbeing.^17^ Studies that have examined the effectiveness of digital health interventions to improve emotional outcomes for patients with HF or other cardiovascular diseases have predominantly focussed on mobile health interventions.^18,19^ A systematic review of smartphone interventions for comprehensive cardiac rehabilitation and HF rehabilitation and management found mHealth interventions feasible and effective in improving quality of life.^18^ However, findings are mixed, with Antypas et al. reporting no statistically significant difference for a mobile intervention in relation to secondary outcomes of anxiety and depression.^20^ Further, small sample sizes were reported. High-quality randomised controlled trials (RCTs) are needed to test whether digital health interventions can improve emotional wellbeing in patients with HF.

The Enhanced HF Care program, a digital health intervention, seeks to improve care coordination with a proactive approach to monitoring and managing emotional and physical wellbeing. The proposed program will achieve this through: (i) recognising the interaction between emotional and physical wellbeing; (ii) harnessing existing community resources, such as GPs; (iii) supporting patient empowerment through offering education and self-help strategies; (iv) providing a mechanism for tailored self-help and stepwise care; (v) using clinically derived algorithms to offer care recommendations; and (vi) increasing the probability of a care partnership between hospital providers, community health, the patient and their support persons.

*Aims:* To determine using a RCT among patients hospitalised with ADHF, the effectiveness and cost-effectiveness of a cardiovascular wellbeing program in (i) improving emotional and physical wellbeing (co-primary outcomes), and (ii) decreasing healthcare utilisation (secondary outcome).

*Hypotheses:* Relative to control participants, intervention participants will have: higher emotional and physical wellbeing scores (co-primary outcomes) 1- and 6-month post-recruitment; and attainment of this increase will be cost-effective, with intervention participants having lower health service utilisation.

## Methods

### Study design and setting

Prospective randomised open blinded end-point design.^21^ A two-arm RCT with participants randomly allocated to receive the intervention (an online enhanced community care program for HF: ‘Enhanced HF Care’) or usual care. Up to 570 patients will be recruited prior to discharge from two hospitals (one metropolitan and one rural) in the Hunter New England local health district. Located within New South Wales, Australia, this region covers more than 130,000 km^2^ and has a population of approximately 910,000. An estimated 55% of the population is in regional or rural areas. The trial was prospectively registered via the Australian New Zealand Clinical Trials Registry: ACTRN12622001289707. Issue date: 4 October 2022.

### Sample

#### Eligibility criteria

*Inclusion criteria:* Patients eligible to participate are those hospitalised with ADHF (including all ejection fraction classifications) being discharged from participating hospitals to home; aged 18 years or older; English speaking; with access to, and ability to utilise, a device with internet; and able to provide informed consent. In 2020, Australian data indicated that 78% of those aged 65 + years had used smartphones to access the internet in the past 6 months, with online activities (including telehealth) substantially increasing throughout the COVID-19 pandemic.^22^

*Exclusion criteria:* Ineligible patients are those without access to a device with internet, and who are unable to provide written informed consent.

#### Recruitment

Prior to hospital discharge, the cardiac research nurse will identify (via daily patient lists) and invite all potentially eligible patients to participate. The nurse will confirm eligibility, provide verbal and written study information and seek the patient's written informed consent to participate via an iPad. Patients will be informed that they do not have to make an immediate decision on participation and can consider the information materials, before deciding whether they would like to participate. If this is the case, the research nurse will visit the patient again in 24 h’ time to answer any questions they might have and seek their consent for participation. If the patient is discharged during this period, they will be unable to participate.

#### Randomisation

Immediately after providing informed consent to participate in the RCT, participants are asked to complete a baseline survey via an iPad. Upon survey completion, the patient will be randomised to either (i) usual care or the (ii) Enhanced HF Care intervention, using permuted blocks, stratified by recruitment site. The allocation sequence is generated by an independent statistician at the Clinical Research Design and IT and Statistical Support Unit of the Hunter Medical Research Institute, and the randomisation schedule is embedded in the patient baseline survey. Group allocation will be created with blocks of sizes two and four selected randomly.

*Blinding:* It is not possible to blind trial participants or cardiac nurses to allocation. Only intervention group participants will be given access to the online enhanced care program via unique link. Research nurses will be aware of trial allocation as they will be involved in supporting intervention group participants to access and use the intervention. Outcome assessors and data analysts will be blinded to allocation.^21^ While there is a small risk that the use of small block sizes could lead to subversion of the randomisation process, the permutation of the different blocks sizes, as well as the fact that the recruiting nurses are not aware of the blocks sizes, with the randomisation sequence performed externally by an independent statistician, will all in aide minimising this risk.

### Control

The usual care (control) group will receive standard care with involvement from cardiology and allied health teams which may differ by site. This includes an initial call within 2 weeks post-hospital discharge to educate and assess patients in regard to their understanding of their condition and treatment, a medication reconciliation and symptom management. Patients are recommended to have a follow-up appointment with their GP 1-week post-discharge and encouraged to formulate an action plan, and an appointment 1 month later with their cardiologist. Further support is offered in relation to exercise, lifestyle modification and palliative care depending on the patient's condition and prognosis. One- and 6-monthly data collection will obtain post-discharge care details including the use of health services.

### Intervention

**Figure 1.**
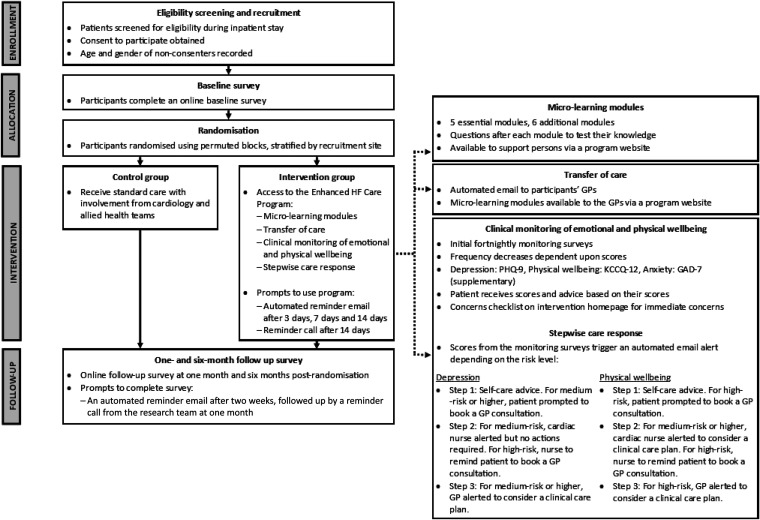
Intervention flowchart.

Participants allocated to this group will receive usual care, plus the Enhanced HF Care program. See Figure 1 for Intervention Flowchart. The cardiac nurse will verbally explain the program to participants. Participants will also receive written detailed instructions on how to use the Enhanced HF Care program.

The Enhanced HF Care program provides patients with relevant health education, regular screening for depression, anxiety and physical wellbeing, and an automated, algorithm-generated stepped care approach to ‘triage’ areas of concern. Via Enhanced HF Care, patients will be prompted to monitor depression and clinical outcomes and offered tailored self-care advice. The clinical team will emphasise the importance of adhering to the guideline-informed care recommendations provided via the program, to achieve the best therapeutic outcomes. While there are barriers to the uptake of digital interventions, several strategies will increase the probability of intervention use. These include: an automated email/SMS from the cardiologist sent to the patient introducing the intervention, as a prescribed element of follow-up care; a reminder email/SMS if the patient has not accessed the program after 3 days; follow-ups and prompts for program use; and integration of the program into the patients support network (GPs and support persons). For example, participants are encouraged to share the educational resources (micro-learning modules) with their support person/s. GPs are also able to access these resources. For participants whose screening identifies them as being at medium risk or higher, GPs will be alerted to consider a clinical care plan. Participants also receive an alert to encourage them to book a consultation with their GP. The scope of the GP's examination is (a) to verify and further assess the severity of depressive symptoms when alerted by the algorithm for stepwise care and (b) to review the adequacy of current treatment intervention and consider any further course of actions as necessary; this may include adjusting medications, referral to a psychiatrist for specialist input, directing the patient for an inpatient or outpatient management via multidisciplinary mental health team depending on the urgency, severity and risks.

The program will include:

*Micro-learning modules:* Videos and written resources embedded within a series of short learning units, i.e. micro-learning modules. All patients will be instructed to complete five essential modules which contain core information and actions for the post-discharge period. These modules cover the following topics: What HF is; symptoms to watch out for; medication management; managing the physical aspects of HF; and how to recognise and manage depression. Additional modules covering optional areas of interest for patients will be offered, including: How to prepare for medical procedures; how to recognise and manage anxiety; physical activity; key health professionals for HF; questions to ask their healthcare team; and strategies to plan for their future. The modules include new content along with links to the National Heart Foundation's existing videos and written resources.^23^ Multiple choice questions for each module are also included for patients to test their knowledge. Participants will be encouraged to share these educational resources with their support person, if available. If micro-learning modules have not been completed within 3 days of being provided access to the program, reminder prompts (email/SMS) will be sent to the patient, with another reminder at 7 days, and again at 14 days, followed by a call from the research team.

*Transfer of care*: An automated email from the cardiologist will be sent to the patients’ GPs recommending the GP to be actively involved in the program. The email will provide GPs with access to the micro-learning modules to refresh their knowledge regarding the nature, treatment and outcomes of HF.

*Clinical monitoring of emotional and physical wellbeing**:*** The Enhanced HF Care program will allow patients to complete monitoring items, using extensively validated measures.

- *Depression*: Patient Health Questionnaire (PHQ)-2, with those scoring ≥3 completing the PHQ-9.^24^ The PHQ-9 is a reliable and accurate self-administered tool commonly used to screen for depression.^24–26^ A score of ≥10 has been found to have an 88% sensitivity and an 88% specificity for major depression.^24^
- *Anxiety*: Generalised Anxiety Disorder (GAD)-2, with those scoring ≥3 completing the GAD-7.^27^ The GAD-7 is an efficient and valid screening tool for assessing GAD severity in clinical practice and research.^27^ A cut-off point of ≥10 has been found to have a sensitivity and specificity >0.80 for anxiety disorders.^27^
- *Physical wellbeing*: Kansas City Cardiomyopathy Questionnaire (KCCQ-12).^28^ The KCCQ-12 has been validated as a reliable and responsive measure of health status in people with congestive HF.^28^

Patients will complete the monitoring survey fortnightly in the first month, post-discharge. Thereafter, the frequency of monitoring will decrease dependent upon the patient's self-reported indication of emotional and physical wellbeing. For example, patients who score low risk for depression on both surveys in the first month will change to monthly depression screening thereafter. Patients who indicate a moderate/high risk for depression, in the first screening instance, will be prompted 1 week later to check whether they have followed the recommendations provided (e.g. accessing self-help strategies, making an appointment with their GP). Fortnightly surveys will continue until those with moderate/high risk depression indicate a reduction in symptoms (See algorithm for stepwise care below).

**Figure 2.**
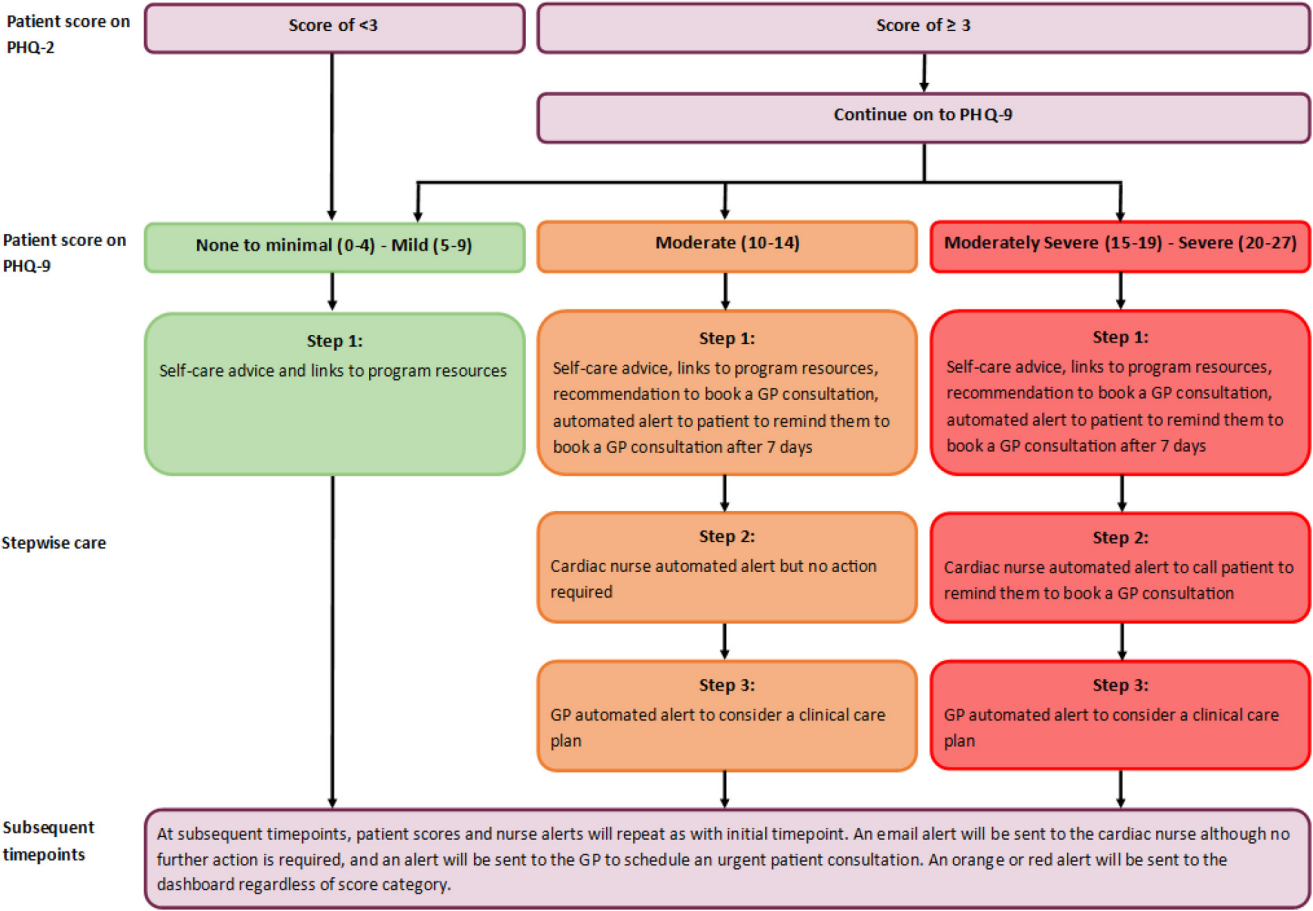
Intervention algorithm for depression stepwise care, based on the Patient Health Questionnaire (9-item).

**Figure 3.**
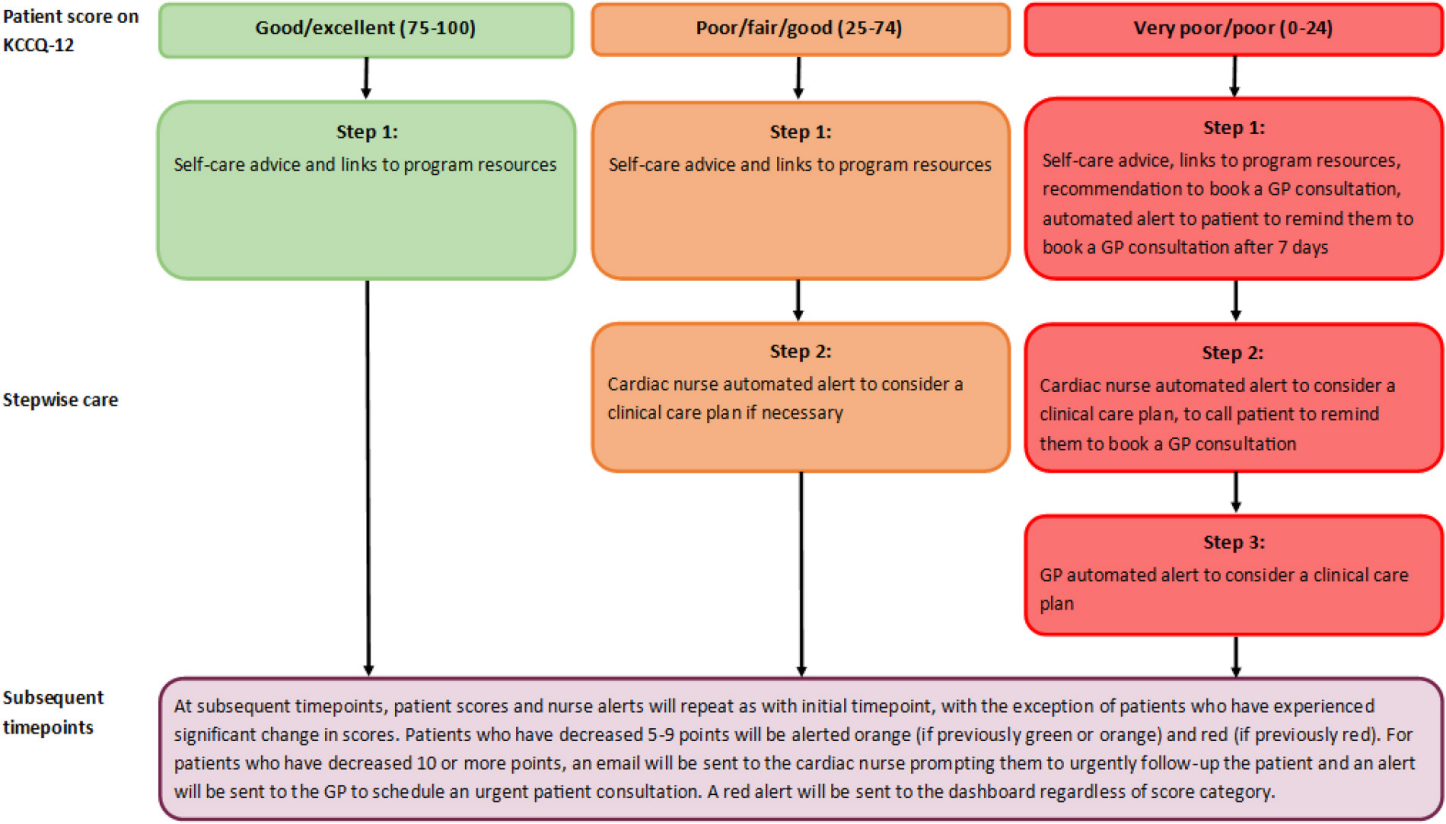
Intervention algorithm for physical wellbeing stepwise care, based on the Kansas city Cardiomyopathy Questionnaire.

*Algorithm for stepwise care:* Stepwise care cost-effectively and efficiently provides the least resource-intensive system, with more intensive support offered as needed.^29^ An algorithm will generate tailored stepwise support depending on the depression and physical clinical monitoring data to ‘triage’ areas of concern (see Figures 2 and 3). Alerts will be sent to the cardiac nurse dashboard, using a traffic light colour system to indicate level of urgency. Data for anxiety will be used to supplement depression and physical care monitoring but does not have an associated stepwise care algorithm. The dashboard is managed using REDCap electronic data capture tools hosted at the Hunter Medical Research Institute, on secure servers which are both physically and virtually secured. REDCap (Research Electronic Data Capture) is a secure, web-based software platform designed to support data capture for research studies, providing (1) an intuitive interface for validated data capture; (2) audit trails for tracking data manipulation and export procedures; (3) automated export procedures for seamless data downloads to common statistical packages; and (4) procedures for data integration and interoperability with external sources.^30,31^

- *Step 1. Self-care:* For low-level risk (e.g. PHQ-9 scores 0–9 and KCCQ-12 scores 75–100) (green alert), self-help strategies will be provided to the patient via encouraging the participants to review their micro-learning module/s. For medium- and high-level risk PHQ-9 scores and high-risk KCCQ-12 scores (orange and red alerts), the survey results will also recommend them to book a GP consultation. An automated prompt will be sent to the patient after 7 days to remind them to use the self-help strategies and book a consultation with their GP.
- *Step 2. Cardiac team dashboard notification:* For low-level risk (green alert), no action will be required. For medium-level risk (orange alerts) PHQ-9 scores, the nurse is alerted but no further action is required. For high-risk (red alerts) PHQ-9 scores, the nurse will be prompted to follow-up with the patient via telephone to remind them to arrange an urgent consultation with their GP. For medium- and high-level risk (orange and red alerts) KCCQ-12 scores, the nurse is suggested to review and consider in their clinical care plan. The nurse will investigate the patients’ psychosocial and/or physical needs and then consult with the cardiovascular and other team members, if necessary, to determine the next phase of care. For high-level risks (red alert), the nurse is also alerted to follow-up with the patient via telephone to remind them to arrange an urgent consultation with their GP. The dashboard will also include variables extracted from medical records for this project. These will include: diagnoses; treatment received; whether the patient has been prescribed common pharmacological treatments for HF; details of the medication regime. This includes any admission and readmission details and pathology and imaging data. This will enable the cardiac nurses on the research team to view this data and the patient-reported data (collected via REDCap surveys monitoring of emotional and physical wellbeing) together on the dashboard, to review and assess any alerts received. Contact with the participants in relation to these alerts will be logged in REDCap.
- *Step 3. GP notification:* For medium- to high-risk PHQ-9 scores (orange and red alerts) and high-risk level KCCQ-12 scores (red alerts), the online program will: notify the patient to attend a GP consultation; send an email to the GP indicating the patient's issue of concern (e.g. high levels of depression); and request the GP's reception staff schedule an urgent patient consultation. The study will also recommend the appropriate proposed treatment actions for each level of depression severity, ranging from no action to immediate initiation of pharmacotherapy and expedited referral to a specialist.^32^ The nurse will also receive an alert to fax the information to the GP if they feel it is necessary.

In addition, participants will also have access to a supplementary Concerns Checklist that they can access anytime they have a symptom or concern. An algorithm will provide specific advice and instructions based on the participant's reported concerns.

### Data collection

The co-primary outcomes are the physical and emotional domains of the Minnesota Living with Heart Failure Questionnaire (MLHFQ), which will be measured at baseline (in hospital prior to discharge), 1 and 6 months. This data collection will allow examination of the short- and long-term impacts of the Enhanced HF Care program. The online baseline survey will be completed in hospital prior to discharge using REDCap. The follow-up surveys will also be completed via a REDCap online survey and participants will be sent an email reminder with the survey link if they do not complete the survey within 2 weeks. Participants will receive up to three $10 grocery eGift Cards in appreciation of their time. A $10 eGift Card will be emailed to them upon completion of each main survey: the first one they will complete in hospital, the 1-month follow-up survey, and the 6-month follow-up survey.

*Primary outcome:* The MLHFQ will be measured at baseline, 1- and 6-month post-recruitment to allow examination of short- and long-term impacts of the intervention. Monitoring measures (e.g. KCCQ-12) differ from the primary outcome measure (MLHFQ) for two reasons. First, some relevant mental health health-related quality of life (HRQL) subdomains (e.g. anxiety and depression) are not well addressed in KCCQ-12.^33^ For example, the KCCQ-12 includes three items on physical limitations, four items on symptom frequency (swelling, fatigue, shortness of breath), two items on QOL (limited enjoyment, satisfaction) and three items on social limitations (hobbies, work, friends), whereas the MLHFQ scale includes both emotional and physical domains. The second reason was to ensure that monitoring measures differ from the primary outcome measure (MLHFQ) to avoid contamination between clinical monitoring throughout the intervention and the primary research outcomes. The MLHFQ attains overall HRQL scores with emotional and physical subscales. It has strong psychometric properties and is sensitive to change, with nearly all scales showing ≥ 0.80 on the responsiveness parameters for patients who have improved.^34^ It has shown good internal consistency across the MLHFQ dimensions, with a Cronbach's alpha ranging from 0.79 to 0.94.^35^ The co-primary outcome variables will be compared between treatment arms at 6-month post-recruitment. Using the physical and emotional domains of the MLHFQ as the co-primary outcome measures avoids contamination associated with using the same measure for evaluating clinical progress for intervention participants.

*Secondary outcomes:* Healthcare utilisation. Unplanned hospital readmissions and hospital emergency department presentations. Measured with data from the medical records and via patient self-report survey.

*Cost-effectiveness/Utility Analysis:* Medical Record Number data will be used to attribute individual patient costs based on hospital utilisation. A medical record audit will be conducted for each consenting patient via routinely collected health service records via the Medical Record Number. The following information will be collected via records audit: diagnoses; treatment received; whether the patient has been prescribed common pharmacological treatments for HF; details of the medication regimen; and whether the patient has been referred to a rehabilitation program. The follow-up surveys will also obtain information not available from hospital records, with additional service use captured using a modified version of the Client Services Receipt Inventory^36^ at 1- and 6-month follow-up, including utilisation of primary care, allied health and other community health services and information to estimate productivity losses and time off work.

*Process measures:* A mixed methods process evaluation will follow the Medical Research Council Framework. To assess acceptability, software analytics will automatically record opening and completion of the micro-learning modules. Views about the ease of use, relevance and quality of intervention components will be assessed by study-specific items at the 1- and 6-month follow-ups and via a telephone interview with a randomly selected subset (up to 30) of consenting intervention participants. Items will examine patient use of Enhanced HF Care and aspects of the program they have used the most and whether they have found these to be beneficial. The semi-structured interview schedule will focus on understanding patterns in the data and issues that arise during the study. Each interview audio-recording will be transcribed verbatim. NVivo software will be used to manage data and to assist with analysis. Interviews will be examined using thematic analysis. Specifically, a general inductive approach to content analysis will be employed, whereby codes, categories, or themes will be directly drawn from the data.

### Minimising attrition

Loss to follow-up will be minimised by (i) obtaining multiple contact details for each participant, (ii) reminder email/SMS prompts if a survey response has not been received within 2 weeks and (iii) a reminder phone call from the research team if a response has not been received within 1 month.

### Sample size and statistical analyses

*Sample size:* The power calculation was based on a previous study involving roughly 1200 patients. This study assessed changes in MLHFQ^34^ sensitivity and the minimum clinically important differences. It reported standard deviations of 7.2 (emotional domain) and 9.5 (physical domain) points, and a 0.5 correlation between baseline and follow-up data across both domains.

Anticipating a 30% drop-out rate by the 6-month follow-up, a baseline sample of 285 participants per group will provide 80% power to detect a clinically significant difference of 1.75 points in the MLHFQ emotional domain and 3.5 points in the physical domain after 6 months, while maintaining an overall type 1 error rate of 5%.

The recruitment phase is projected to span 18 months, based on the volume of patients and expected consent rate. With the participating hospitals treating approximately 540 ADHF patients annually, the proposed sample size is feasible*.*

*Statistical analysis:* The primary analysis population will be the intention to treat, defined as all randomised participants. The co-primary outcome variables will be compared between treatment arms using separate linear mixed effects regression models. The models will include: fixed categorical effects for time and treatment group, baseline value of the outcome variable, and their interaction. Stratification variables will be included as fixed effects. The model will account for serial correlations from the repeated measures on the same individuals. Least squared mean differences will be presented, with 95% confidence intervals and *p*-values. Assuming that missing outcomes are random would be unrealistic in this study due to the high death rate. Instead, we will address missing data by assuming it is not randomly missing, using pattern mixture models. These models hypothesise differences in mean outcomes between missing and non-missing data. By adjusting this parameter across a plausible range (tipping point method), we can test how much our conclusions rely on this assumption. To maintain the cumulative probability of a type 1 error at 5%, we will test each outcome in a hierarchical manner. We will only proceed to the second outcome if the hypothesis test for the first one yields a *p*-value less than .05. Testing will commence with the emotional domain, and then proceed to the physical domain.

*Cost-effectiveness/utility analysis:* The cost-effectiveness analysis will include both the cost of implementing the Enhanced HF Care program and the cost of healthcare utilisation (inpatient, outpatient, emergency department, and primary care visits) obtained from patient self-report and hospital admission data. The effectiveness will be measured in terms of reduced utilisation, improved HRQL self-reported through the 21-item MLHFQ and the quality-adjusted life years (QALYs) using a mapping algorithm^37^ that convert the MLHFQ score to the 5-level EuroQoL-5D utility score QALY for the economic evaluation. The algorithm has been successfully validated in a previous study.^38^ Using the trial-based analysis, the joint estimates of costs and effects will be combined in an incremental analysis between two strategies and presented as the point estimate of the mean incremental cost-effectiveness ratio (ICER) for the Enhanced HF Care program versus current practice. The ICER will be calculated as the cost difference divided by the effect difference (QALYs) between the two arms.^39^ Sensitivity analyses will be performed to test the robustness of the results to changes in key parameters and assumptions. The analysis results will be reported in accordance with the Consolidated Health Economic Evaluation Reporting Standards (CHEERS) checklist guidelines.^40^

### Trial status

Recruitment commenced in July 2023. As at 5 April 2024, 65 participants are enrolled.

## Discussion

The development, implementation, and rigorous testing of this multicomponent enhanced care program, if effective and cost-effective, has the potential for profound impact on patients with HF. Providing ongoing emotional and physical support to this high-risk patient group and their supportive others is fundamental for improving care and reducing healthcare utilisation. However, the type of discharge support currently provided for patients with acute HF tends to focus on physical, rather than emotional, aspects of care and is inconsistent across hospitals and healthcare providers.^41^ Most commonly, written information is provided but patient utilisation and comprehension of these materials is not well understood.

Digital solutions offer the opportunity to provide standardised emotional and physical monitoring and support for patients. To date, most studies have instead investigated the effectiveness of digital health interventions in improving behaviour risks for cardiovascular disease.^19,42^ For example, a 2021 systematic review found that internet-based interventions were generally effective.^43^ High-quality RCTs are needed to test whether internet-based interventions can improve emotional wellbeing in patients with HF. The cost-effectiveness of digital health interventions for cardiovascular populations has also been examined with a recent review reporting that all digital health interventions included in their review were cost-effective.^17^ However, studies that have examined the effectiveness of digital health interventions to improve emotional outcomes for patients with HF or other cardiovascular diseases have predominantly focussed on mobile health interventions only. Few published studies have investigated digital health education interventions for managing depression for patients with HF.^44^ The Enhanced HF Care program will harness existing digital health intervention knowledge in the cardiovascular research field and rigorously apply this to provide both emotional and physical monitoring for patients.

Implementation and translation to practice, should the intervention prove successful, would be low cost and readily achievable with most electronic medical records.

## Conclusion

It is expected that Enhanced HF Care will lead to patients appropriately managing emotional and physical symptoms and thereby reducing their mental health burden, as well as the frequency and length of hospitalisations.
